# EPR and pulsed ENDOR study of intermediates from reactions of aromatic azides with group 13 metal trichlorides

**DOI:** 10.3762/bjoc.6.84

**Published:** 2010-08-09

**Authors:** Giorgio Bencivenni, Riccardo Cesari, Daniele Nanni, Hassane El Mkami, John C Walton

**Affiliations:** 1 Dipartimento di Chimica Organica “A. Mangini”, Università di Bologna, Viale del Risorgimento 4, Bologna I-40136, Italy; 2 School of Physics and Astronomy, University of St. Andrews, St. Andrews, Fife KY16 9SS, UK; 3 School of Chemistry, University of St. Andrews, EaStChem, St. Andrews, Fife KY16 9ST, UK

**Keywords:** aluminium, aromatic azides, ENDOR, EPR, gallium, indium

## Abstract

The reactions of group 13 metal trichlorides with aromatic azides were examined by CW EPR and pulsed ENDOR spectroscopies. Complex EPR spectra were obtained from reactions of aluminium, gallium and indium trichlorides with phenyl azides containing a variety of substituents. Analysis of the spectra showed that 4-methoxy-, 3-methoxy- and 2-methoxyphenyl azides all gave ‘dimer’ radical cations [ArNHC_6_H_4_NH_2_]^+•^ and trimers [ArNHC_6_H_4_NHC_6_H_4_NH_2_]^+•^ followed by polymers. 4-Azidobenzonitrile, with its electron-withdrawing substituent, did not react. In general the aromatic azides appeared to react most rapidly with AlCl_3_ but this reagent tended to generate much polymer. InCl_3_ was the least reactive group 13 halide. DFT computations of the radical cations provided corroborating evidence and suggested that the unpaired electrons were accommodated in extensive π-delocalised orbitals. A mechanism to account for the reductive conversion of aromatic azides to the corresponding anilines and thence to the dimers and trimers is proposed.

## Introduction

The number of applications of indium [1–6], gallium [7–11] and other group 13 metal derivatives, as promoters of radical reactions, has been increasing ever since the original work of Baba and co-workers with dichloroindium hydride [12–16]. Parallel to that, organic azides are increasingly used as sources of N-centred radicals, although most such methods also require organotin hydrides [17–23]. In seeking cleaner, less toxic and more efficient synthetic methodology – not reliant on organotin compounds [24–28] – some of us began investigating the reactions of organic azides with dichloroindium hydride [29], allylindium dichloride [30], and other group 13 metal derivatives. These reagents smoothly convert aromatic and aliphatic azides into the corresponding amines, γ-azidonitriles into pyrrolidin-2-imines [29], and δ-azidoesters and chlorides into allylated nitrogen heterocycles [30].

To help in elucidating the mechanisms of these reductions, we used CW EPR spectroscopy and attempted to characterise the reactive intermediates in selected reactions involving gallium trichloride. Surprisingly, we found that treatment of phenyl azide and 4-methoxyphenyl azide with gallium trichloride resulted in strong EPR spectra of long-lived paramagnetic species. By combining the results of product analyses with the results of EPR spectroscopy, we were able to show that persistent radical cations of ‘dimers’ (4-aminodiphenylamines) and ‘trimers’ (4′-phenylamino-4-aminodiphenylamines) were being formed [31]. We have now broadened the scope of this investigation to aromatic azides with a range of functionality. We report here our findings on the behaviour of aromatic azides when treated with the group 13 trichlorides of gallium, indium and aluminium.

## Results and Discussion

### Reaction of 4-methoxyphenyl azide (**2**) with group 13 metal chlorides

**Scheme 1:**
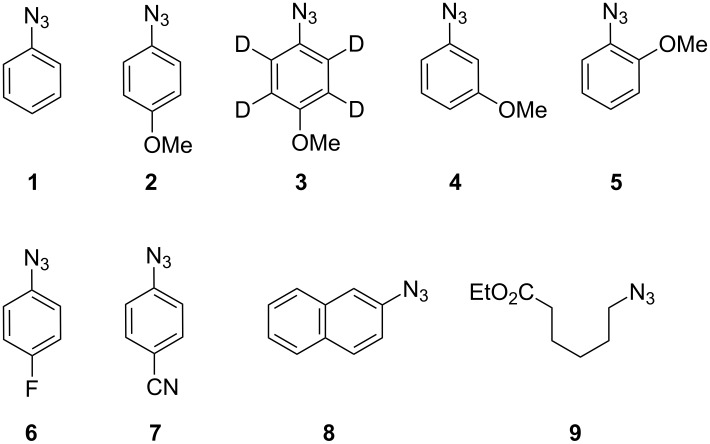
Organic azides studied.

**Scheme 2:**
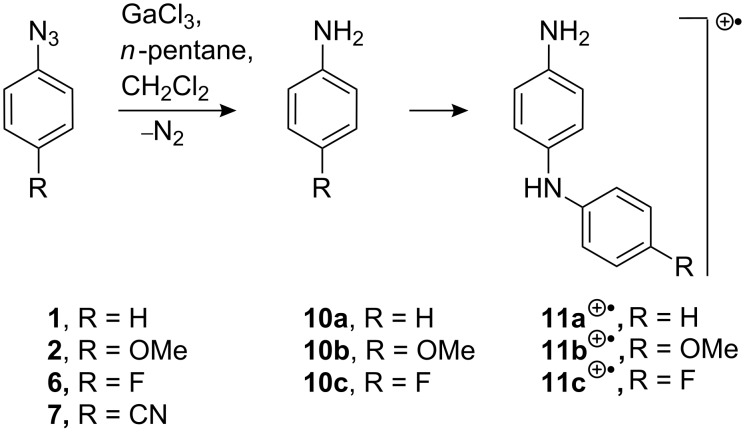
Reaction of 4-substituted-phenyl azides with GaCl_3_.

A set of aromatic azides, each containing an electron-releasing or an electron-withdrawing substituent in the 4-position, was chosen for this study. The position of the substituent was also varied and several other azide types were included (Scheme 1). Each organic azide was reacted with the metal halide in dichloromethane/pentane or acetonitrile solution at rt, and an aliquot (~0.1 mL) was placed in a quartz capillary tube (diam 1 mm), purged with nitrogen for 15 min and transferred to the resonant cavity of an X-band EPR spectrometer. When either AlCl_3_, or GaCl_3_ or InCl_3_ was used, the reaction was accompanied by copious evolution of gas (probably nitrogen) and a deep blue or violet colour usually developed immediately or within a few minutes. In the case of AlCl_3_ the reactions were very vigorous. Previously, we showed that the main product from the reaction of 4-methoxyphenyl azide **2** with GaCl_3_ was 4-amino-4′-methoxydiphenylamine (**11b**, Variamine blue), together with traces of anisole, oxidised derivatives (including 4-(4-methoxyphenylamino)phenol, 4-((4-methoxyphenyl)imino)-cyclohexa-2,5-dienone) and much dark-coloured polymer [31]. The EPR spectrum showed the radical cation of Variamine blue (**11b**^+•^) plus broad signals which we attributed to oligomer and/or polymer radical cations (Scheme 2).

**Figure 1:**
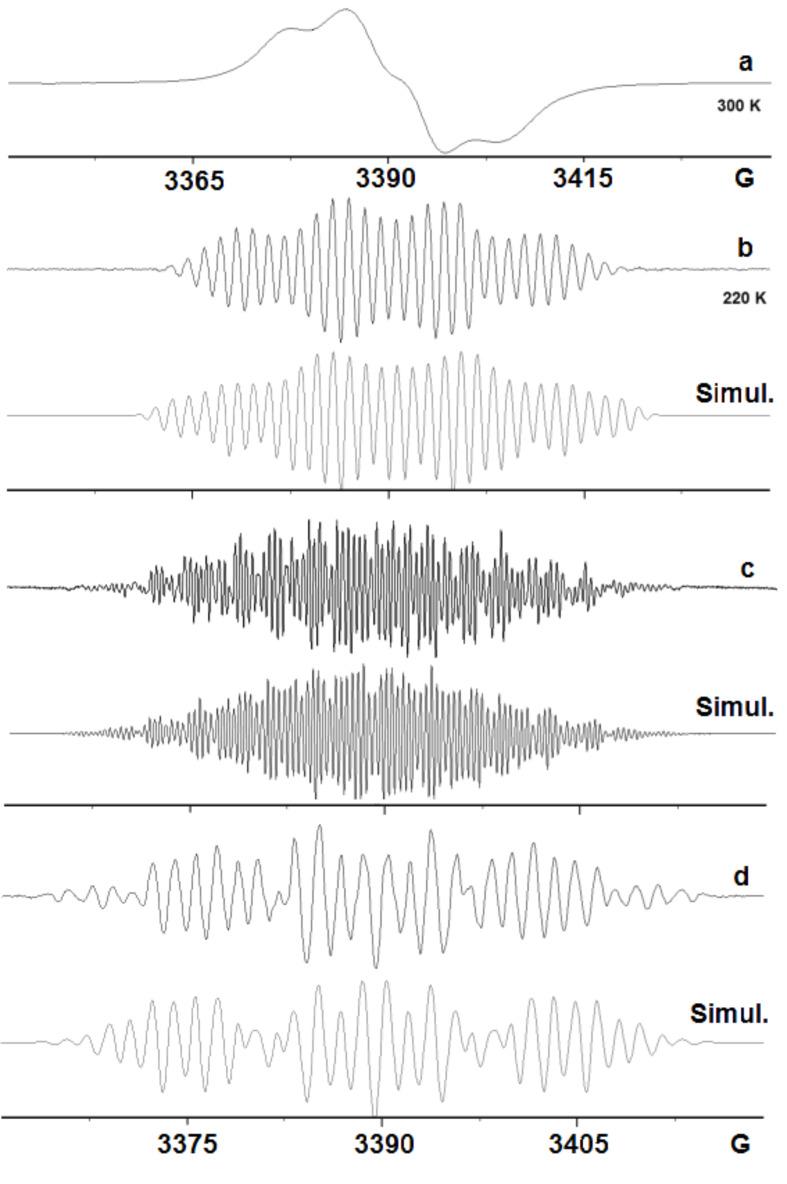
EPR spectra after treatment of azide **2** with MCl_3_. (a) AlCl_3_ in DCM; 1st derivative spectrum at 300 K. (b) AlCl_3_ in DCM; 2nd derivative spectrum at 220 K; below: computer simulation with the parameters listed in Table 1. (c) GaCl_3_ in DCM/pentane at 300 K with the computer simulation below. (d) InCl_3_ in DCM:CH_3_CN; 4:1 at 320 K; computer simulation below.

When anhydrous AlCl_3_ in DCM – instead of the gallium halide – was added to a solution of azide **2**, a vigorous reaction took place. The resulting deep-coloured solution was transferred to the EPR spectrometer and initially the spectrum, Figure 1a, was obtained at 300 K. The broad, poorly resolved signal suggested that the mixture was dominated by polymeric material. However, when the solution was cooled down to 220 K, the well-resolved spectrum, Figure 1b, was obtained. The resolution improvement may be due to the fact that most of the polymer separates from the solution at the lower temperature.

A good computer simulation was achieved by utilising the hyperfine splitting constants (hfs) listed in Table 1. A well-resolved EPR spectrum of **11b**^+•^, generated from **2** with GaCl_3_, is shown in Figure 1c for comparison. Figure 1d shows the experimental spectrum obtained from treatment of **2** with InCl_3_, together with the corresponding computer simulation. Although the three EPR spectra appear different at first sight, the hfs derived from the simulations (Table 1) are actually quite similar. The contrasts in the spectral appearances are mainly the result of different line widths with consequently different resolutions. It is evident that the main species in each case is the radical cation **11b**^+•^. The acceptable agreement between the DFT-computed isotropic hfs of **11b**^+•^ (Table 1) and the experimental data provides additional support for this identification. The small differences in the hfs obtained for the different group 13 metal chlorides can probably be attributed to the different counter ions, solvents and temperatures.

**Table 1:** EPR parameters of ‘dimers’ [ArNHC_6_H_4_NH_2_]^+•^ from treatment of aryl azides with group 13 metal chlorides.^a^

Precursor	MCl_3_/solvent	Radical cation species	1N	1N	(N)H	(N)H_2_	2H	2H	2H	2H	Other

PhN_3_ **1**^b^	GaCl_3_/DCM	**11a**^+•^	4.9	4.9	6.8	5.6	3.1	2.0	1.0	0.6	1H, 1.0
PhN_3_ **1**	AlCl_3_/DCM, ACN	**11a**^+•^	4.5	4.5	6.3	5.0	2.2				
PhN_3_ **1**	DFT^c^	**11a**^+•^	4.3	2.2	−8.4	−4.8	−2.0	−1.8	−1.1	1.0	1H, −2.3
4-MeOC_6_H_4_N_3_ **2**	AlCl_3_/DCM	**11b**^+•^	4.9	4.3	7.3	3.7	1.2	1.2			
4-MeOC_6_H_4_N_3_ **2**^d^	GaCl_3_/DCM	**11b**^+•^	5.2	4.4	7.3	3.8	3.1	2.2	0.8	0.4	
4-MeOC_6_H_4_N_3_ **2**	InCl_3_/DCM 4, ACN 1	**11b**^+•^	5.2	4.0	7.8	3.2	3.2	3.2	1.8		
4-MeOC_6_H_4_N_3_ **2**	DFT^c^	**11b**^+•^	4.2	2.0	−8.1	−4.4	−2.0, −1.6	−1.6, −1.4	−0.8	0.2	MeO, 1.2
4-MeOC_6_D_4_N_3_ **3**	AlCl_3_/DCM		5.9	4.8	6.7	4.9	–	–	–	–	
4-MeOC_6_D_4_N_3_ **3**^d^	GaCl_3_/DCM		5.2	4.4	7.1	3.6	0.66, 2D	0.55, 2D			
3-MeOPhN_3_ **4**^b^	GaCl_3_/ACN	**17a**^+•^	4.3	–	5.7	5.2, 5.2	5.7, 5.7	5.2, 5.7	–	–	
3-MeOPhN_3_ **4**	DFT^c^	**17a**^+•^	3.8	1.1	−7.6	−2.3, −2.0	−7.2, −7.3	2.6, −3.4	−2.3, 1.3	−0.9, −0.2	MeO, −0.1
2-MeOC_6_H_4_N_3_ **5**	GaCl_3_/DCM	**17b**^+•^	4.1	4.1	5.5	5.5					
2-MeOC_6_H_4_N_3_ **5**	DFT^c^	**17b**^+•^	4.0	1.5	−8.1	−3.9	−3.1, −2.3	−1.5, −1.6	0.8, 0.7	−0.3, 0.1	MeO, 0.8
4-FC_6_H_4_N_3_ **6**	HGaCl_2_/ACN, TES^e^	**11c**^+•^	4.4	3.9	4.0	4.0	2.0	2.0	1.0	0.5	1F, 6.6
4-FC_6_H_4_N_3_ **6**	DFT^c^	**11c**^+•^	4.0	2.5	−7.9	−5.5	−1.7	−1.4	−1.4	0.5	4.3^f^
2-NapN_3_ **8**	InCl_3_/DCM 4, ACN 1		3.4	3.4	5.6	2.8	2.8 (1H)				

^a^All *g*-factors were 2.0032 ± 0.0005. Assignments of hfs to specific atoms are tentative and are based on the DFT computations. Note that only the magnitudes and not the signs of hfs can be derived from the EPR spectra. ^b^Treatment of PhN_3_ and 3-MeOC_6_H_4_N_3_ with InCl_3_ gave only very weak and broad unresolved spectra. ^c^DFT computations: geometries optimised to UB3LYP/6-31+G(d,p) then single point calculations with 6-311++G(d,p) basis. ^d^Data from ref [31]. ^e^HGaCl_2_ prepared from GaCl_3_ and Et_3_SiH (TES). ^f^The computed *a*(*F*) varied from 4.3 G, with the 6-311++G(d,p) basis set, to 9.5 G with the DGDZVP basis set.

**Figure 2:**
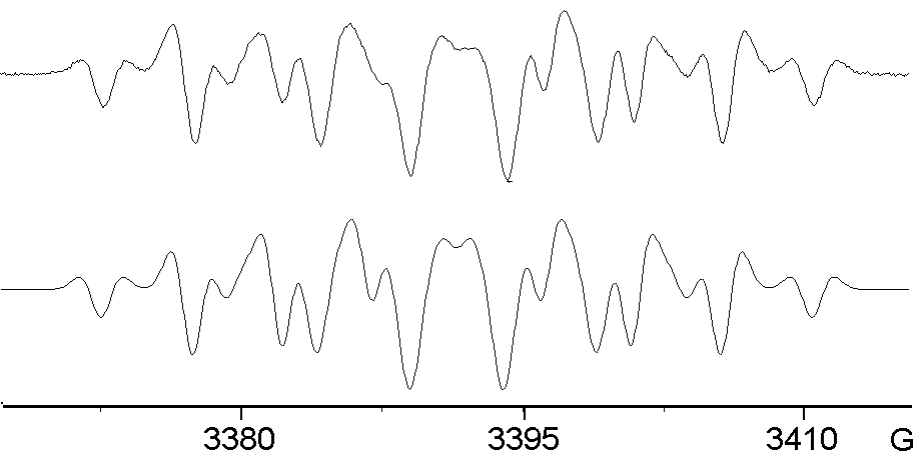
EPR spectrum after treatment of tetra-deuterated azide **3** with AlCl_3_. Top: 2nd derivative spectrum at 290 K in DCM. Bottom: computer simulation using the hfs from Table 1.

The 4-methoxy-tetradeuterio-azide **3** was also treated with AlCl_3_ in DCM, and the resulting spectrum and simulation are shown in Figure 2.

The unpaired electron interacts with two non-equivalent N-atoms, a single comparatively large H-atom and a pair of equivalent H-atoms. The spectrum obtained previously on treatment of **3** with GaCl_3_ was better resolved (Table 1) [31]. However, it is clear that the same ‘dimer’ species was formed with AlCl_3_, probably having picked up the NH and NH_2_ hydrogen atoms from the solvent. The line width of the spectrum with AlCl_3_ was ca. 0.7 G. Therefore, it is not surprising that hfs from aromatic ring D-atoms were not resolved. Again, differences in the hfs of the spectra from AlCl_3_ and GaCl_3_ can be attributed to the different counter ions.

### Reactions of phenyl azide and 4-substituted-phenyl azides with group 13 metal chlorides

**Figure 3:**
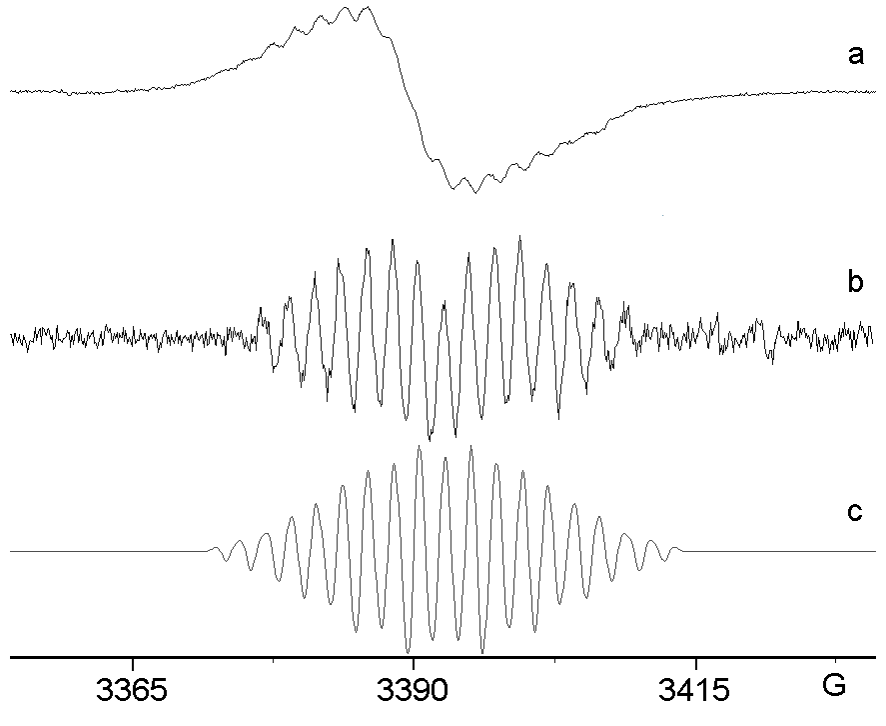
EPR spectra after treatment of azide **1** with AlCl_3_. (a) 1st derivative spectrum in DCM at 280 K. (b) 2nd derivative spectrum after digital removal of residual broad central component. (c) computer simulation.

Azides **1**, **6** and **7** were chosen to vary the electronic properties and leaving group abilities of 4-substituents. We showed previously that treatment of phenyl azide **1** with GaCl_3_ gave well-resolved spectra of 4-aminodiphenylamine radical cation (**11a**^+•^, the dimer) and of the trimer under different reaction conditions [31]. On treatment with InCl_3_, **1** gave little sign of reaction. No colour developed and no EPR spectra were obtained. However, a vigorous reaction took place between **1** and AlCl_3_ with nitrogen evolution and development of a deep blue colour. The EPR spectrum, Figure 3a, was dominated by a broad component, probably due to polyaniline type material, together with some fine structure. The second derivative spectrum at low modulation amplitude discriminated against the broad signal, and spectrum, Figure 3b, was obtained after digitally removing the residual broad component.

The hfs were similar to those of **11a**^+•^ (Table 1, entry for **1** with GaCl_3_) except that the smaller hfs were not resolved. Minor differences in the magnitudes of the hfs can be attributed to the different counter ions. The trimer radical cation was not observed, but clearly a contribution from this species could be hidden under the broad component.

No reaction of 4-azidobenzonitrile **7** with InCl_3_, GaCl_3_ or AlCl_3_ was observed and no paramagnetic species were detected by EPR spectroscopy. It appears the electron-accepting property of the CN group inhibited the coupling process at some stage. It is also worth mentioning that, as expected, aliphatic azides such as ethyl 5-azidopentanoate **9** did not react in the same way either. Treatment of **9** with InCl_3_ or GaCl_3_ led to gas evolution but no colour developed and no paramagnetic species could be detected.

**Figure 4:**
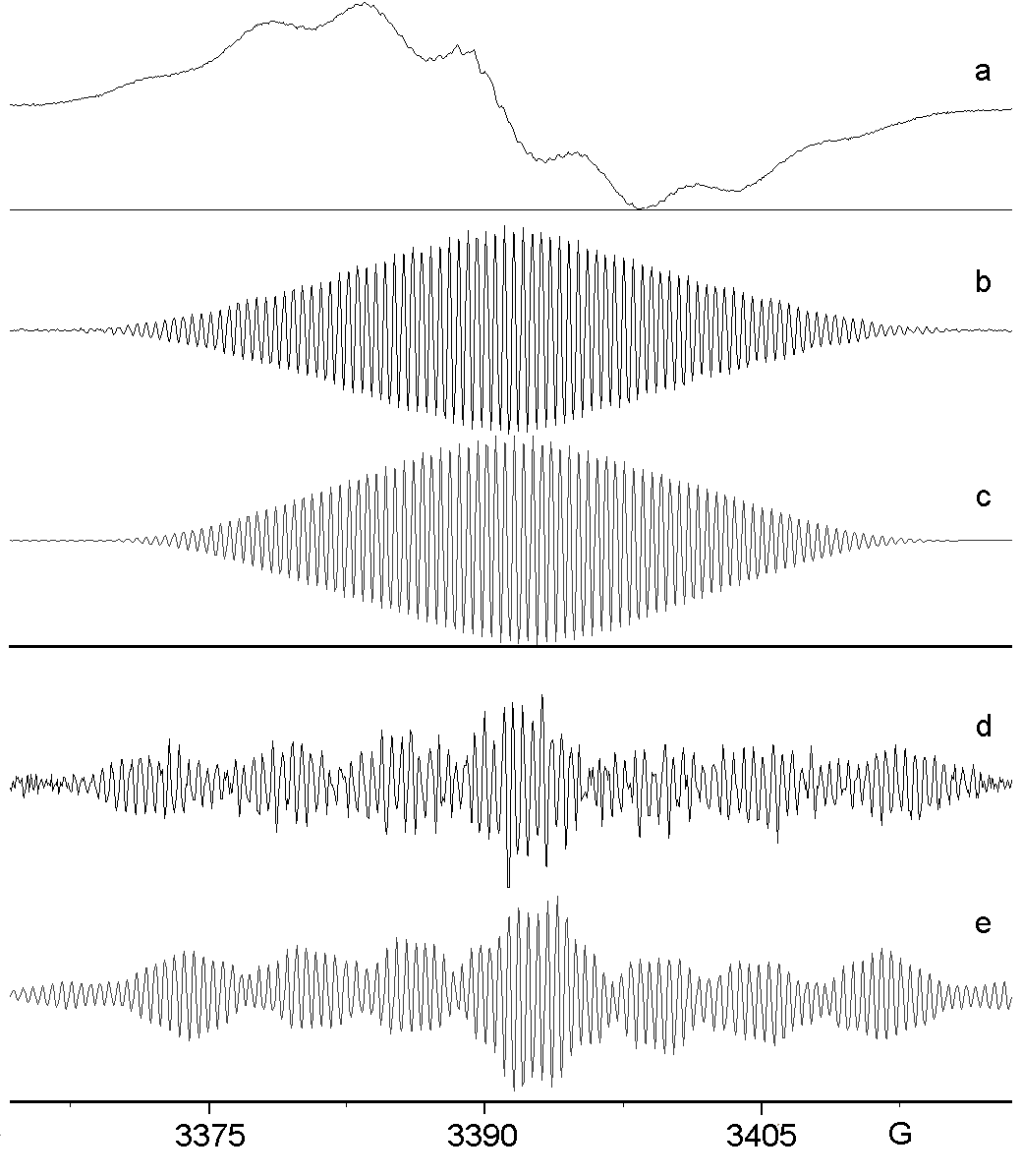
EPR spectra after GaCl_3_ and InCl_3_ reactions of azide **6**. (a) 1st derivative spectrum from **6** and GaCl_3_ in DCM at 300 K. (b) 1st derivative spectrum of dimer (**11c**^+•^) from **6** and HGaCl_2_ in CH_3_CN at 300 K. (c) computer simulation of (b) with hfs of Table 1. (d) 1st derivative spectrum of trimer (**19**^+•^) from **6** and InCl_3_ in DCM at 300 K. (e) computer simulation of (d) with hfs of Table 2.

Very interesting results were obtained from reactions of 1-azido-4-fluorobenzene (**6**). When **6** was treated with GaCl_3_ in DCM, a deep blue-violet colour developed and the spectrum was dominated by a broad feature, Figure 4a, although underlying fine structure was evident. When dichlorogallium hydride, prepared from GaCl_3_ and Et_3_SiH in CH_3_CN, was used to promote the reaction, a beautifully resolved spectrum resulted, Figure 4b.

The good simulation of this spectrum, Figure 4c, enabled the hfs shown in Table 1 to be determined. Comparison of these hfs with those of the other species in Table 1 supports the identification of this intermediate as the corresponding dimer cation **11c**^+•^, containing a single F-atom. The DFT computation on the dimer hfs gave satisfactory agreement (Table 1), with the possible exception of the *para*-F hfs. However, DFT-computed *a*(*F*) values varied from 7.2, to 7.1, to 9.5 and 4.3 G with 6-31G(d), 6-31+G(d,p), DGDZVP and 6-311++G(d,p) basis sets, respectively. This spread indicates the comparative unreliability of the DFT spin density computations for F-atoms in these cations.

Treatment of **6** with InCl_3_ in DCM led to the usual broad signal from oligomeric and polymeric species superimposed on a spectrum with much narrower lines. On recording the spectrum with a smaller modulation amplitude, and digitally removing the residual broad feature, the spectrum shown in Figure 4d resulted. This is obviously a different species from that of Figure 4b and, after many trials, a satisfactory simulation was obtained, Figure 4e. The derived hfs are presented in Table 2 and they clearly correspond to a trimer, also probably containing a single F-atom, i.e. **19b**^+•^.

**Table 2:** EPR hfs of ‘trimer’ species {[ArNH]_2_C_6_H_4_NH_2_}^+•^ from treatment of aryl azides with group 13 metal chlorides.^a^

Precursor	MCl_3_/solvent or DFT	Trimer radical cation	N	N	N(H_2_)	(N)H_2_	(N)H	(N)H	H-rings	Other

PhN_3_ **1^b^**	GaCl_3_/DCM	**19a**^+•^	5.0	4.9	3.0	6.5	4.9	2.1	2.1 (3H) 1.0 (3H)	
PhN_3_ **1**	DFT^c^	**19a**^+•^	5.4	3.4	2.0	−3.0	−7.8	−4.8	−1.6 (4H) −1.0 (3H) <±0.6 (6H)	
4-FC_6_H_4_N_3_ **6**	InCl_3_/DCM	**19b**^+•^	5.5	5.5	2.6	6.2	7.2	4.5	2.2 (1H) 1.5 (2H) 0.5 (6H)	2.2 (1F)
4-FC_6_H_4_N_3_ **6**	DFT^d^	**19b**^+•^	3.7	2.1	1.5	−3.2	−7.2	−4.2	−1.3 (4H) −0.6 (4H) <0.6 (4H)	1.5 (1F)
2-MeOC_6_H_4_N_3_ **5**	InCl_3_/DCM	**18**^+•^	4.8	4.8	4.8	5.1	5.1	5.1	–	
2-MeOC_6_H_4_N_3_ **5**	HGaCl_2_^e^/CN	**18**^+•^	4.5	4.5	3.0	4.1 1.7	5.5	5.5	1.7 (1H) 0.5 (4H)	
2-MeOC_6_H_4_N_3_ **5**	DFT^c^	**18**^+•^	4.4	3.9	1.5	−2.0 −1.6	−6.6	−5.1	−2.0 (1H) −1.7 (2H) −1.1 (1H) <0.7 (8H)	0.4 (CH_3_O)

^a^All *g*-factors 2.0032 ± 0.0005, assignments of hfs to specific atoms are tentative and are based on the DFT computations. Note that only the magnitudes and not the signs of hfs can be derived from the EPR spectra. ^b^Data from ref [31]. ^c^DFT: UB3LYP/6-31G(d). ^d^DFT computations: UB3LYP/6-31+G(d,p) then single point calculation with 6-311++G(d,p) basis. ^e^HGaCl_2_ prepared from GaCl_3_ and Et_3_SiH (TES).

It seems clear that the MCl_3_ reactions with aromatic azides entail a progression from the aniline XC_6_H_4_NH_2_, to the dimer XC_6_H_4_NHC_6_H_4_NH_2_, to the trimer XC_6_H_4_NHC_6_H_4_NHC_6_H_4_NH_2_, thence to oligomers and eventually polyaniline type polymers X[C_6_H_4_NH]*_n_*C_6_H_4_NH_2_. Some polymer radical cation was always observed by EPR spectroscopy, but whether dimer or trimer or oligomer dominated the spectrum depended on a delicate balance between solvent, metal halide and other factors.

### Reactions of 2-methoxy- and 3-methoxyphenyl azides with group 13 metal chlorides

**Scheme 3:**
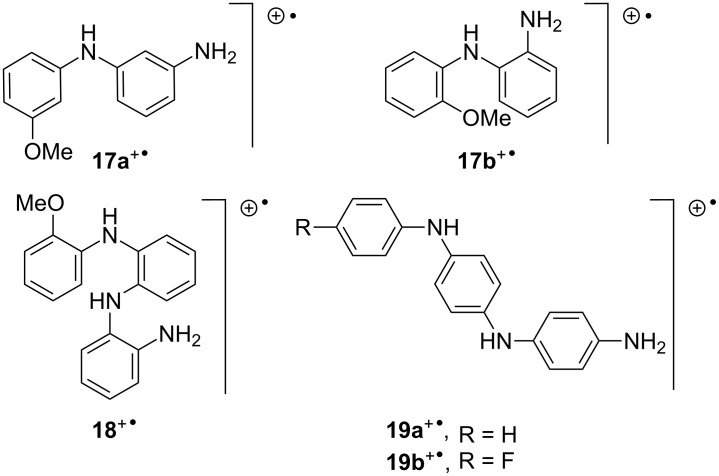
Dimer and trimer radical cations.

Aromatic azides **4** and **5** were chosen to investigate how the position of the MeO substituent influenced the reaction. Treatment of the 3-methoxy precursor **4** with InCl_3_ or HInCl_2_ gave only very weak and broad EPR spectra. However, reaction of **4** with GaCl_3_ in CH_3_CN gave a strong EPR spectrum and the hfs derived from the computer simulation are presented in Table 1. The comparatively large line width (~0.9 G) did not permit the resolution of small hfs from aromatic ring H-atoms. For the same reason, the hfs from the second N-atom were not resolved. However, it is clear that this species is probably a ‘dimer’ although the connectivity of the angular structure **17a**^+•^ is somewhat different from that of the 4-aminodiphenylamines derived from the 4-substituted phenyl azides (Scheme 3).

**Figure 5:**
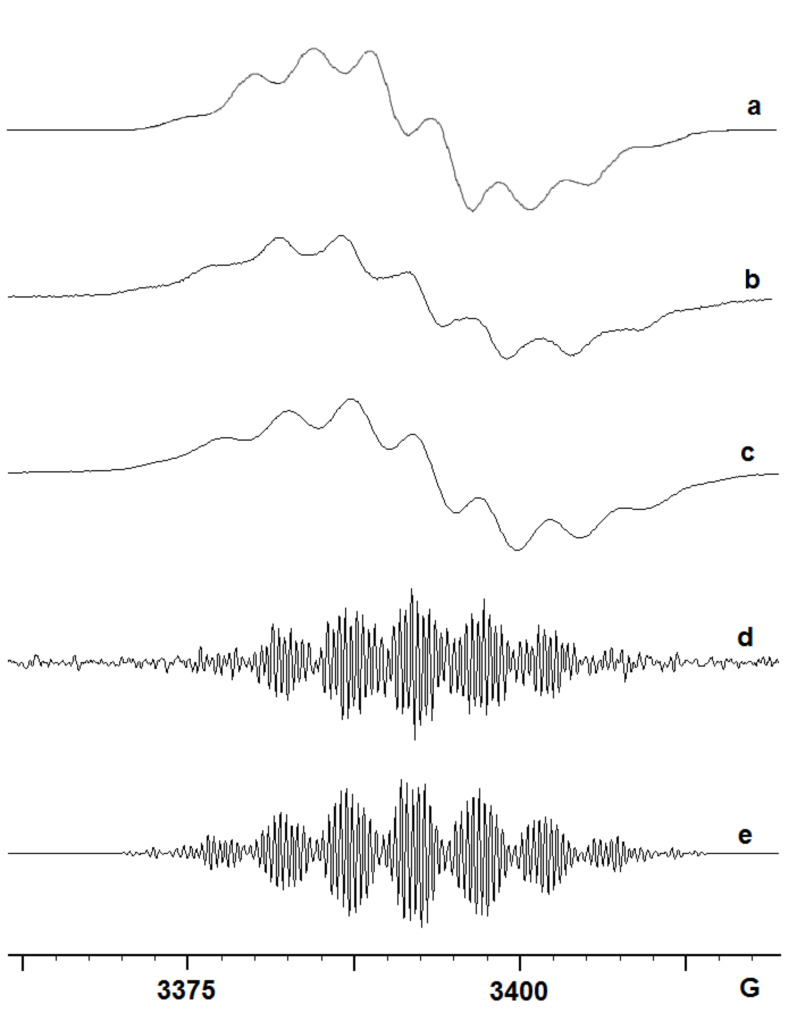
EPR spectra after GaCl_3_- and InCl_3_-promoted reactions of 2-methoxyphenyl azide **5**. (a) 1st derivative spectrum of **17b**^+•^ from **5** with GaCl_3_ in DCM. (b) 1st derivative spectrum of **17b**^+•^ from **5** with HInCl_2_ in THF at 300 K. (c) 1st derivative spectrum of **18**^+•^ from **5** with InCl_3_ in DCM at 300 K. (d) 1st derivative spectrum of **18**^+•^ from **5** with HGaCl_2_ in CH_3_CN at 300 K. (e) computer simulation of (d).

Treatment of the 2-methoxy precursor **5** with GaCl_3_ in DCM, or with HGaCl_2_ in CH_3_CN, gave essentially the same strong spectrum, see Figure 5a and Supporting Information. The hfs derived from the simulations (Table 1) suggest that this is also a dimer type radical cation **17b**^+•^. However, with the passage of time a central peak began to appear in this spectrum. When **5** was treated with HGaCl_2_, prepared from GaCl_3_ and Et_3_SiH, the species with a central peak dominated the spectrum, Figure 5b. Treatment of **5** with InCl_3_ in DCM or with HInCl_2_ in THF (prepared from InCl_3_ and DIBAL-H) also gave rise to a spectrum of this same species, Figure 5c. A well-resolved spectrum of this species was obtained by treatment of **5** with HGaCl_2_ prepared with Et_3_SiH in CH_3_CN, Figure 5d. The hfs derived from the computer simulation, Figure 5e and Table 2 show the presence of three N-atoms and of four H-atoms with sizeable hfs that can probably be attributed to NH or NH_2_ groups. Thus, this species is almost certainly a ‘trimer’ although this will necessarily have an angular structure **18**^+•^ rather than the linear type structure of the trimers from 4-subsitituted azides such as **19**^+•^ (Scheme 3).

The results from azides **4** and **5** showed that the position of the MeO substituent in the phenyl azides was not critical. The reactions with gallium and indium promoters proceeded along similar lines to that of phenyl and 4-substituted phenyl azides to give dimers, trimers and polymers.

**Figure 6:**
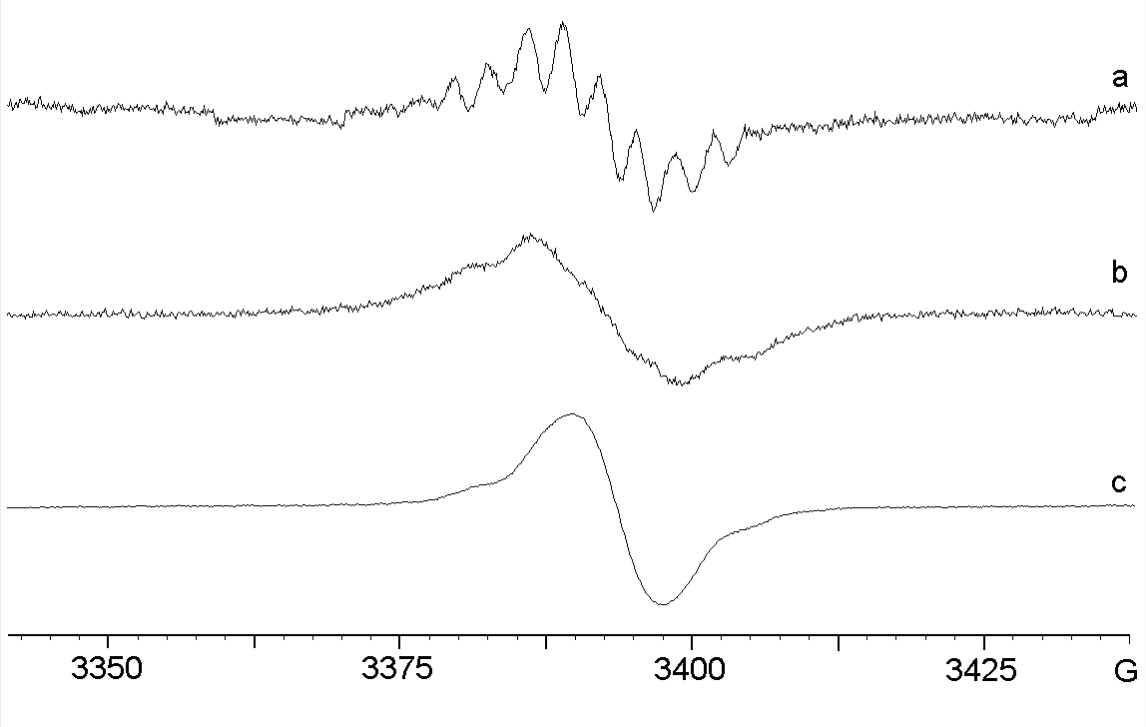
EPR spectra after In-, Ga- and Al-promoted reactions of azide **8**. (a) intermediate from InCl_3_ treatment of **8** at 260 K in DCM and CH_3_CN (4:1). (b) spectrum from GaCl_3_ treatment of **8** at 300 K in DCM/pentane. (c) spectrum from AlCl_3_ treatment of **8** at 300 K in DCM.

The spectra obtained on treatment of 2-azidonaphthalene **8** with InCl_3_, GaCl_3_ and AlCl_3_ are shown in Figure 6a, Figure 6b and Figure 6c, respectively.

The broad signal in Figure 6c shows that polymerisation dominated the reaction with AlCl_3_. Similarly, the main broad feature in Figure 6b suggests that polymerisation was again dominant in the reaction with GaCl_3_. The comparatively well-resolved species observed in the InCl_3_-promoted reaction, Figure 6a, was well simulated on using the parameters shown in Table 1. The data show that the unpaired electron interacted with two N-atoms, two (N)H_2_ atoms, one (N)H-atom and one other H-atom; other splittings were not resolved. The magnitudes of the hfs are somewhat smaller than those of analogous atoms in the dimer from **1**. This is exactly as would be expected from the greater extent of aromatic delocalisation in a dimer from **8**. Clearly, however, more than one isomer is possible.

### Pulse ENDOR spectrum of the intermediate from 4-fluorophenyl azide **6**

Pulsed ENDOR experiments, based on the ESE effect, were carried out on the frozen solution from azide **6** at 50 K. The echo signal was created by the microwave pulse sequence, and an rf pulse was applied during the ‘mixing period’, which corresponded to the time *T* in the Davies ENDOR sequence [32]. The rf pulse drove the nuclear spin transitions, which led to a change in the ESE intensity. The ENDOR signal was therefore measured by monitoring the ESE intensity while the rf frequency was varied. In the case of an *S* = ½ system coupled with a nucleus with nuclear spin *I* = ½, the Davies ENDOR spectrum consists of two lines at the nuclear resonance frequencies ν_α_ and ν_β_, which correspond to the transitions associated with the electron spin manifolds *M*_s_ = +½ and *M*_s_= −½, respectively. If the Larmor frequency (ν_n_) of the nucleus in question is larger than the hyperfine interaction, then the resonance frequencies are given by:

[1]
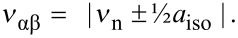


If ν_n_ is less than ½*a*_iso_, the frequencies are then given by:

[2]
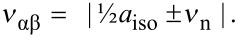


An additional complication arises if the nuclear spin is >½, which adds another term describing the nuclear quadrupole interaction in the above equations [33]. In frozen solution all orientations of the paramagnetic species are observed and therefore an anisotropic ENDOR spectrum is expected. The latter is more complex and requires a detailed understanding of the anisotropy of the system. The above equations are not suitable for such a situation and a more complete resonance condition that considers all the orientations is needed. In the case of a system with *I* = ½ the parameter *a*_iso_ in Equation 1 and Equation 2 is replaced by *A*_i_ (i.e. one of the principal components of the hyperfine tensor).

**Figure 7:**
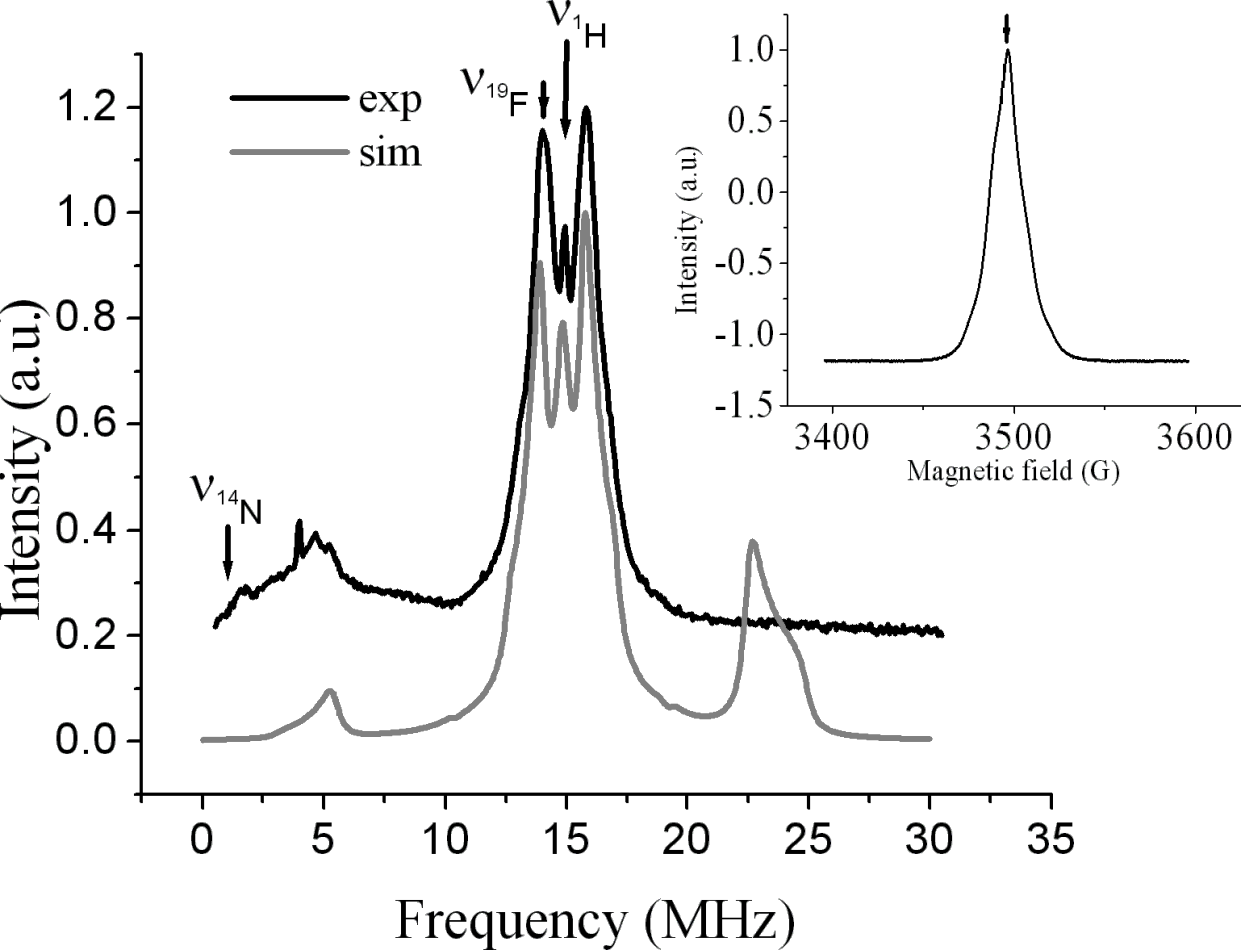
Experimental and simulated Davies ENDOR spectrum after the Ga-promoted reaction of azide **6** recorded at 50 K. The inset shows the field-swept EPR spectrum with an arrow indicating the magnetic field position of the ENDOR experiment.

The Davies ENDOR spectrum from the species derived from the 4-fluoroazide **6** sample at 50 K is shown in Figure 7. The inset shows the ESE-EPR spectrum, with an arrow indicating the magnetic field position at which the ENDOR experiment was performed. The ENDOR spectrum shows powder pattern lineshapes, as expected for frozen solutions, due to the anisotropic hyperfine interactions. Two main features cover the whole spectrum; a powder pattern centred about the ^1^H Larmor frequency and a second broad signal located at lower frequency and spread over 8 MHz width.

The lack of resolution encountered in the ENDOR spectrum makes an unequivocal analysis difficult. Therefore, our ENDOR simulation was based mainly on the CW EPR results. A simulated spectrum is displayed in Figure 7, and a deconvoluted version is in the Supporting Information together with one chosen set of ENDOR hyperfine tensor parameters. Almost all the ^1^H hyperfine splittings fit well within the ENDOR spectra, but it is worth noting, as mentioned above, that a satisfactory simulation was only achieved by assuming an anisotropic lineshape of the hyperfine couplings. Extra weak hyperfine couplings, unresolved in the CW EPR, were also included in the simulation. These probably correspond to hyperfine coupling in polymer which was undoubtedly present. The broad feature at low frequency is related to a mixture of fluorine (^19^F) and nitrogen (^14^N) contributions. The anisotropy and the unresolved nuclear quadrupole of the nitrogen couplings make the spectra difficult to interpret. Each ^1^H contributes three sets of peaks to the spectrum times the number of ^1^H’s present. This represents an enormous number of lines in one spectrum. Obviously, they cannot all be assigned from this broad unresolved powder pattern. Almost axial tensors were assumed (see Supporting Information). However, it should be noted that it may well be possible to simulate these spectra with other parameter sets. The experimental Davies ENDOR data support the CW EPR data in confirming the magnitudes of the hyperfine couplings and the nitrogen interactions. Further pulse techniques such as electron spin echo envelope modulation (ESEEM) and its multidimensional extension Hyscore would be required to get more insight into the nitrogen contribution. Regarding the ^19^F contribution; only the low-frequency part of the ^19^F coupling fits well with the experimental data. The ^19^F high-frequency line in our simulation is not consistent with the experimental spectrum, which suggests that the latter might be highly asymmetric. Such situations have been previously reported in other studies where it was shown that this could be related to the relaxation time. Sometimes relaxation processes can lead to a partial saturation in the nuclear transitions such that the observed signal is the result of a transition in one manifold only [34]. Partial saturation may explain the absence of the ^19^F high-frequency line in our spectrum.

### DFT computations of radical cation properties

Quantum chemical calculations were carried out with the Gaussian 03 programme package [35–36]. Density functional theory with the UB3LYP functional was employed. The equilibrium geometries were fully optimised with respect to all geometric variables, no symmetry being assumed either with the 6-31+G(d,p) basis set (dimers) or with the 6-31G(d) basis set (trimers). Isotropic EPR hfs were derived from computed Fermi contact integrals evaluated at the H- and N-nuclei. The hfs were taken directly from the Gaussian output files and are shown in Table 1 and Table 2.

**Figure 8:**
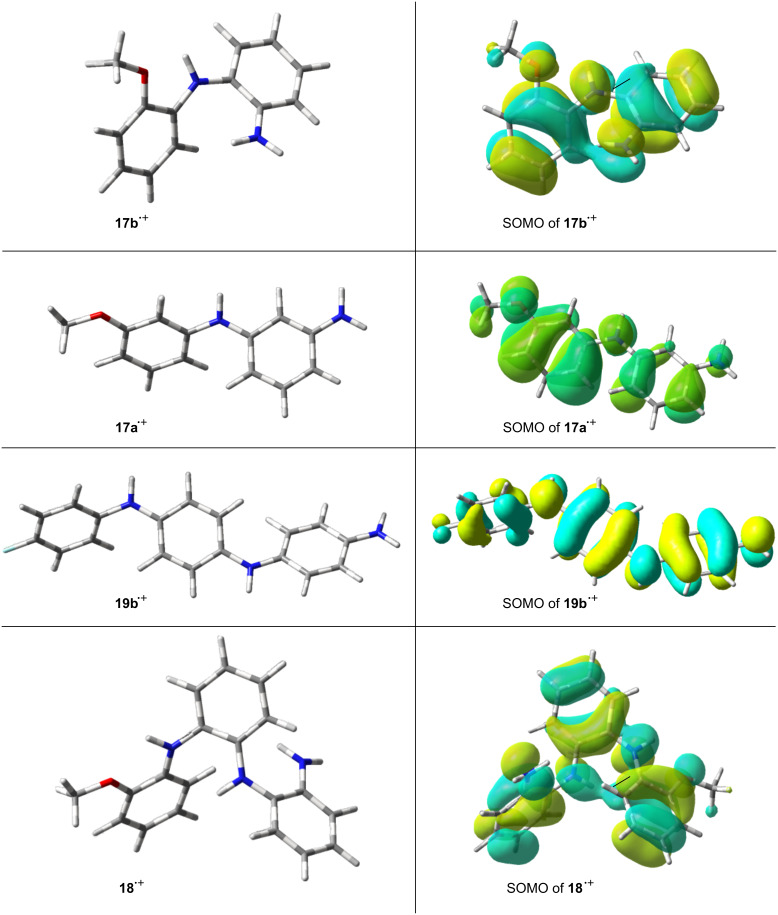
DFT structures and SOMOs for dimer and trimer radical cations.

The optimum structures of the radical cations **17a**^+•^ and **17b**^+•^ their associated SOMOs are shown in Figure 8.

The C–NH bond lengths in radical cation **17a**^+•^ (1.40 and 1.38 Å) and **17b**^+•^ (1.38, 1.39 Å) indicated significant double bond character. The CNC angles in **17a**^+•^ and **17b**^+•^ were 128.9 and 131.6°, showing significant widening from trigonal. The aromatic rings in all the structures were twisted significantly out of co-planarity. As might be expected on steric grounds, this increased in the dimers as the substitution site moved from the 4- to the 3- to 2-position. For example, the computed dihedral angles between the rings increased from 26.8 to 30.7 to 39.0 in the series 4-MeO-**11b**^+•^, 3-MeO-**17a**^+•^ and 2-MeO-**17b**^+•^, respectively. It seems that a compromise was reached in which the repulsive steric interaction between substituents of neighbouring rings was balanced against the stabilising effect from conjugation of the π-systems. The SOMOs depicted in Figure 8 show that there was still sufficient orbital overlap in the linear and angular dimer and trimer radical cations to support lengthy π-systems extending over all the rings and N-atoms. This is in accordance with the EPR spectroscopic data, that show extensive delocalisation of the unpaired electron in dimer and trimer radical cations. The computed hfs in Table 1 and Table 2 show reasonable correspondence with the experimentally observed values.

## Conclusion

Literature reports show that anilines can easily be oxidised to the corresponding resonance-stabilised radical cations, which can couple with more aniline to afford very persistent radical cation dimers [37–38]. The generation of these radical cations depends critically on the reaction conditions, in particular on the degree of protonation, which can facilitate electron transfer (ET) [39–40]. It has also been reported that electrochemical oxidation of aromatic amines can generate the same radical cations which can polymerise giving oligo- and poly-anilines [41]. In view of the fact that product analyses [31] identified aniline amongst the products from **1** and anisole amongst the products from **2**, it seems probable that the aromatic amines are the precursors of the dimer and trimer species.

**Scheme 4:**
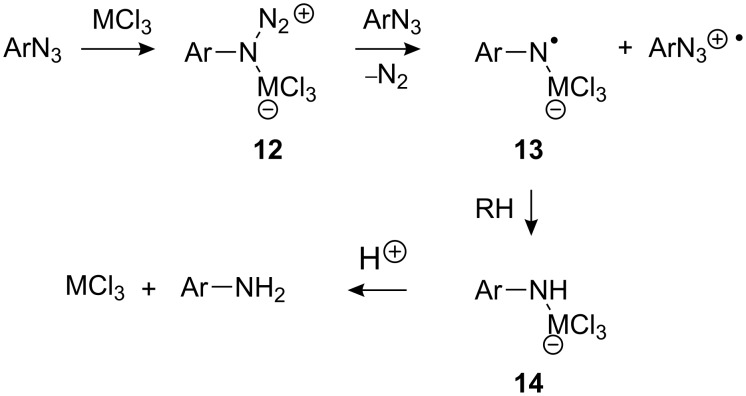
Possible mechanism of formation of aromatic amines.

A possible mechanism for production of anilines from the aromatic azides is set out in Scheme 4. Coordination of the metal halide to the starting azide should produce the Lewis base–acid adduct **12** that could undergo reduction by ET from more azide to afford, after nitrogen loss, the metal-coordinated aminyl radical **13** together with the ArN_3_^+•^ radical cation. Aminyl radical **13** could then abstract an H-atom from solvent RH (or from HMCl_2_ when the metal hydrides were used) with the production of metal-coordinated amine **14**. The latter can then pick up a proton to produce an aromatic amine and regenerate the metal halide. The reason 4-azidobenzonitrile **7** did not react with any of the group 13 metal chlorides may well be that the ET step **12** → **13** was inhibited by the presence of the electron acceptor CN group.

**Scheme 5:**
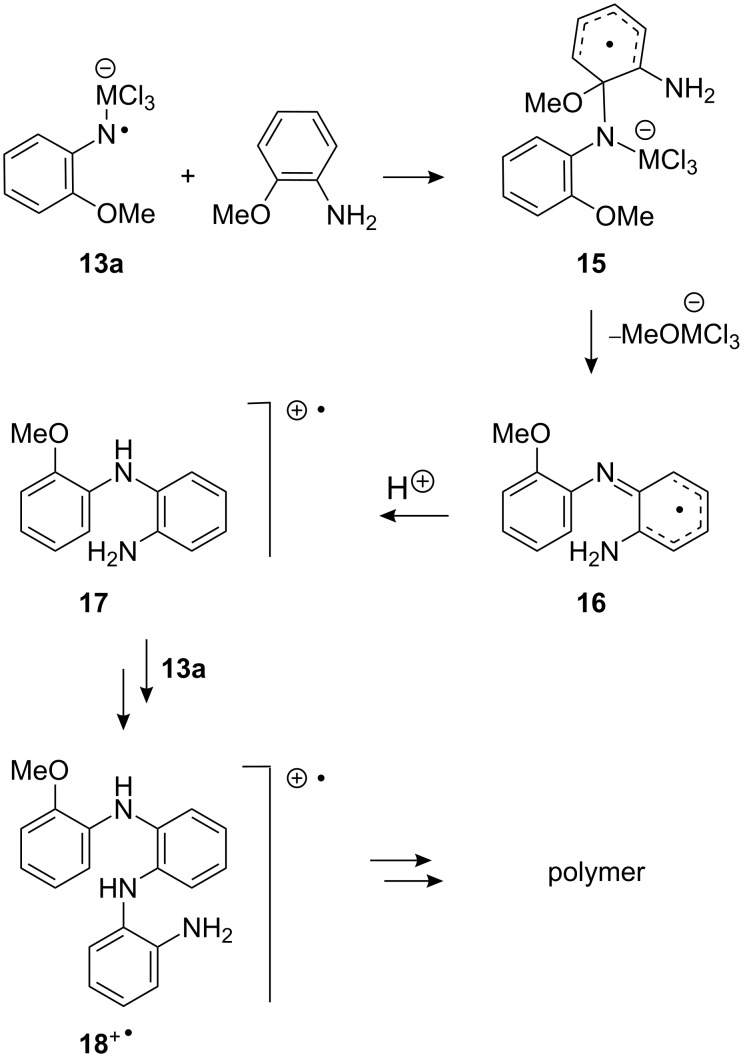
Possible mechanism for dimer and trimer formation.

Several mechanisms have been proposed in the literature for the formation of ‘dimers’ from anilines. These include [42–44]: (i) initial formation of the radical cation ArNH_2_^+•^ which then couples with more aniline and forms the 4-aminodiaryl amine radical cation after loss of HX and (ii) formation of the aniline radical ArNH^•^, which couples with ArNH_3_^+^, ArNH_2_ or ArNH_2_^+•^. A plausible mechanism for formation of the dimer and trimer radical cations we observed is shown in Scheme 5 for the case of 2-methoxyaniline.

*Ipso* attack by radical **13a** on the aniline would lead to the production of delocalised radical **15**. Elimination of MeOMCl_3_^−^ would then yield radical **16**, which, on protonation, would afford the observed long-lived dimer radical cations **17**^+•^. Of course, proton transfer could occur earlier in the reaction, such that coupling takes place with the anilinium cation instead. Trimer **18**^+•^ could be produced by coupling of **17**^+•^ with more **13a** followed by a similar sequence of steps. The trimer could then grow into oligomer and polymer by a succession of such coupling reactions.

In general, the aromatic azides appeared to react most rapidly with AlCl_3_ but this reagent tended to generate much polymer. InCl_3_ was the least reactive group 13 halide such that no reaction was observed with PhN_3_ and very little reaction occurred with the 3-methoxyazide **4**. GaCl_3_ and HGaCl_2_ were the best promoters. The dimers were the main products from reactions of excess **1** and **2** with GaCl_3_ such that the process could have synthetic potential. In general, the GaCl_3_**-** and HGaCl_2_**-**promoted reactions were also best for spectroscopic studies because they gave the most intense and well-resolved spectra of [ArNHArNH_2_]^+•^ and/or {[ArNH]_2_ArNH_2_}^+•^ with the narrowest line widths.

## Experimental

**EPR and ENDOR spectroscopy.** EPR spectra were obtained with a Bruker EMX X-Band 10/12 spectrometer fitted with a rectangular ER4122 SP resonant cavity and operating at 9.4 GHz with 100 kHz modulation. An aliquot (~0.1 mL) of the reaction mixture from each aromatic azide and the metal chloride in CH_2_Cl_2_/pentane or CH_3_CN solution was placed in a 1 mm o.d. quartz capillary tube, de-aerated by bubbling nitrogen for 20 min and transferred to the resonant cavity. Spectra were examined at several temperatures but generally best resolution and signal intensity were obtained at around 300 K. Most of the EPR spectra were recorded with 2.0 mW power, 1.0–0.2 *G*_pp_ modulation intensity and a gain of ~10^6^. In all cases where spectra were obtained, hfs were assigned with the aid of computer simulations using the Bruker SimFonia and NIEHS Winsim2002 software packages.

Pulsed EPR and ENDOR were performed using a pulsed EPR X-band spectrometer (Bruker Elexsys E580) equipped with a Dice-ENDOR accessory, a radio frequency (rf) amplifier and a dielectric-ring ENDOR resonator (Bruker EN4118X-MD-4-W1). Samples were maintained at 50 K using liquid helium in an Oxford CF-935 cryostat. Field-swept electron spin echo (ESE) spectra were recorded using a two-pulse ESE sequence while ESE-ENDOR experiments were carried out using Davies three-pulse sequence π-*T*-π/2-τ-π-echo with a selective rf pulse of variable frequency applied during time *T*. The pulse lengths used were 128 and 256 ns for π/2 and π respectively, and 10 μs for the π-rf pulse. ENDOR data were processed and simulated using the EasySpin package (freeware from http://www.easyspin.org/).

**DFT calculations.** All computations were done with the Gaussian 03W programme package (Version 6.1.0.0) [35]. Geometries were optimised at the UB3LYP/6-31+G(d,p) level [45] (dimers) and the UB3LYP/6-31G(d) level (trimers) and single point calculations at these geometries with a triple zeta quality basis set (6-311++G(d,p)) were used to predict isotropic EPR hfs. The DGTZVP basis set, similar to that recommended by Schäfer et al. [46], was also employed for some computations.

**General procedure for the reaction of aryl azides with indium trichloride.** The starting azide (1 mmol) was added at 0 °C to an acetonitrile solution of indium trichloride (1.1 mmol) in DCM (4 mL) and stirred for 5 min at 0 °C. Gas was evolved and the solutions took on a dark blue or violet colour. The resulting solutions were rapidly transferred into a quartz capillary tube and purged with nitrogen for few minutes. The tube was sealed and placed in the EPR resonant cavity. Spectra were recorded at several different temperatures. Some samples were photolysed with a 500 W super pressure Hg arc.

**General procedure for the reaction of aryl azides with dichloroindium hydride.** The starting azide (1 mmol) was added at 0 °C to an acetonitrile solution of dichloroindium hydride (1.1 mmol), generated in situ by stirring under an argon atmosphere anhydrous indium trichloride (243 mg, 1.1 mmol, previously dried by heating at 130 °C under argon for 1 h) and triethylsilane (177 μL, 1.1 mmol) in ACN (4 mL) for 5 min at 0 °C [47]. The resulting solution was rapidly transferred into a quartz capillary tube and nitrogen was bubbled inside for few minutes. The tube was sealed and placed in the EPR cavity. Spectra were recorded at several different temperatures. Some samples were photolysed with a 500 W super pressure Hg arc. Selected samples were given an aqueous work-up with NaHCO_3_ followed by extraction with diethylether. In each case the corresponding aromatic amine was identified by comparison with literature data.

**General procedure for the reaction of aryl azides with AlCl_3_.** Aluminium trichloride (1.1 mmol) was dried under reduced pressure at 25 °C for 1 h. Then DCM (3 mL) was added and a DCM solution of the azide (1 mmol in 1 mL) was introduced at rt. Gas was evolved, sometimes violently, and dark blue or violet colours developed. The resulting solution was then transferred to a capillary quartz tube and purged with nitrogen. The capillary was sealed and several EPR spectra were run at different temperatures. Product analysis was performed by quenching the reaction with an aqueous solution of NaOH and extracting with DCM. The mixtures were analysed by GC–MS and, when possible, by ^1^H NMR and ^13^C NMR spectroscopy.

**General procedure for the reaction of aryl azides with GaCl_3_.** A pentane solution of gallium trichloride (0.55 mL of 0.5 M; 0.28 mmol) was added under a nitrogen atmosphere to a DCM solution of the azide (0.25 mmol in 4 mL) at rt. Gas was evolved and an intense blue or violet colour developed. The resulting solution was then transferred into a capillary quartz tube and purged with nitrogen. The capillary was sealed, and the sample was analysed by EPR spectroscopy at several different temperatures. Products analysis was performed as above.

**Ethyl 5-azidopentanoate (9)** [48] was prepared by treatment of the corresponding alkyl bromide with sodium azide in DMSO [49]; IR (ν_max_, CHCl_3_), 1718 (CO) and 2092 (N_3_) cm^−1^; ^1^H NMR (400 MHz) δ 1.24 (t, *J* = 7.2 Hz, 3H), 1.54–1.77 (m, 4H), 2.32 (t, *J* = 6.9 Hz, 2H), 3.28 (t, *J* = 6.6 Hz, 2H), 4.13 (q, *J* = 7.2 Hz, 2H).

Aromatic azides **1**–**8** were prepared by standard diazotisation of the corresponding anilines followed by treatment with sodium azide, and were identified by comparison with literature data: phenyl azide (**1**) [50], 1-azido-4-methoxybenzene (**2**) [48], 1-azido-3-methoxybenzene (**4**) [51], 1-azido-2-methoxybenzene (**5**) [48], 1-azido-4-fluorobenzene (**6**) [48], 4-azidobenzonitrile (**7**) [52] and 2-azidonaphthalene (**8**) [53]. 2,3,5,6-Tetradeuterio-4-methoxyphenyl azide (**3**) was prepared by diazotisation of 2,3,5,6-tetradeuterio-4-methoxyaniline, derived in turn from the reaction of 3,5-dideuterio-4-methoxyaniline hydrochloride with boiling D_2_O for 4 days in a sealed tube [31].

## Supporting Information

Supporting information features general procedures, EPR spectra from azides **4** and **5,** deconvolution of ENDOR spectrum from azide **6**, Cartesian coordinates for DFT-computed structures of dimer and trimer radical cations.

File 1 EPR and pulsed ENDOR study of intermediates from reactions of aromatic azides with group 13 metal trichlorides
