# Supplementary material for: EPR and pulsed ENDOR study of intermediates from reactions of aromatic azides with group 13 metal trichlorides
Source: Beilstein J Org Chem. 2010 Aug 9;6:713–25. doi: 10.3762/bjoc.6.84 (PMC2956481; doi:10.3762/bjoc.6.84)
Supplement: File 1 — EPR and pulsed ENDOR study of intermediates from reactions of aromatic azides with group 13 metal trichlorides [file Beilstein_J_Org_Chem-06-713-s001.pdf]

Supporting Information

for

EPR and pulsed ENDOR study of intermediates from reactions of aromatic azides with group 13 metal trichlorides

Giorgio Bencivenni[1], Riccardo Cesari[1], Daniele Nanni[1], Hassane El Mkami[2], and John C. Walton*[3]

Address: [1]Dipartimento di Chimica Organica "A. Mangini", Università di Bologna, Viale del Risorgimento 4, Bologna I-40136, Italy; [2]School of Physics and Astronomy, University of St. Andrews, St. Andrews, Fife KY16 9SS, UK and [3]School of Chemistry, University of St. Andrews, EaStChem, St. Andrews, Fife KY16 9ST, UK

Email: John C. Walton - jcw@st-and.ac.uk

* Corresponding author

Table of contents

General procedures S2
Preparation of deuteriated azide **3** S2–S3
EPR spectra from 2- and 3-methoxyphenyl azides (**4**, **5**) S3-S4
ENDOR spectrum from **6** with deconvolution S4
Cartesian coordinates for DFT calculated structures of dimer and trimer radical cations S5–S9
References S10

General procedures. ^{1}H NMR spectra were recorded at 400 MHz using $CDCl_3$ solvent as reference and/or internal deuterium lock. ^{13}C NMR spectra were recorded at 75.5 MHz using the PENDANT sequence and internal deuterium lock. The chemical shifts for all NMR spectra are expressed in parts per million to high frequency of TMS reference. Coupling constants (*J*) are quoted in Hz and are reported to the nearest 0.1 Hz. The IR spectra were obtained with an FT-IR system. Solids were run as nujol mulls and liquids were run as thin films on NaCl plates. Mass spectra were recorded at low resolution and high-resolution (HR) using a CI, time-of-flight, orthogonal acceleration spectrometer coupled to a GC system. Electrospray mass spectra (ESMS) were recorded on a high performance orthogonal acceleration reflecting TOF mass spectrometer, coupled to an HPLC instrument. Only major peaks are reported and intensities are quoted as percentages of the base peak. TLC was carried out using Polygram silica plates (0.2 mm with 254 nm fluorescent dye) and the components were observed under ultraviolet light (254 nm/365 nm). Column chromatography was performed on silica gel (40–63 μm, Fluorochem). Hexane, DCM, ethyl acetate and toluene were used as supplied. Pyridine was dried with KOH. Nitrogen gas was dried (NaOH, $CaCl_2$, 4 Å molecular sieves) prior to use.

General procedure for EPR analysis of reactions of aryl azides with gallium trichloride. A pentane solution of gallium trichloride (0.5 M, 1.1 equiv) was added under a nitrogen atmosphere to a dichloromethane solution of the azide (1 equiv in 4 mL) at rt. The resulting solution was then transferred in a capillary quartz glass tube and purged with nitrogen for 15 min. The capillary was then sealed and the sample transferred to the resonant cavity of the EPR spectrometer. Several spectra were recorded at different temperatures and over a period of several hours (sometimes days). Product analysis was performed by quenching the reaction with an aqueous solution of NaOH and extracting with dichloromethane. The extract was analysed by GC–MS and, when possible, by ^{1}H NMR and ^{13}C NMR spectroscopy. Product identification was performed by comparison with literature data.

2,3,5,6-Tetradeuterio-4-methoxyphenyl azide (3). Gaseous HCl was bubbled into a solution of 3,5-dideuterio-4-methoxyaniline (8.5 mmol) in diethyl ether to give the corresponding hydrochloride salt which was filtered, transferred to a glass tube and

dissolved in D_2O (6 mL). The tube was sealed and the mixture was boiled for 2 days, the exhausted D_2O was then removed by distillation and replaced with fresh D_2O (6 mL). The new mixture was boiled for two days. The reaction was neutralised with aq. NaOH and extracted with dichloromethane to give the 2,3,5,6-tetradeuterio-4-methoxyaniline (7.48 mmol, 88%). ^{1}H NMR (400 MHz) δ_H 3.36 (bs, 2H), 3.74 (s, 3H); ^{13}C NMR (100 MHz) δ_C 55.7 (CH_3), 114.4 (t, J = 24.2 Hz, CD), 116.0 (t, J = 24.2 Hz, CD), 139.7 (C), 152.7 (C). TOF MS EI$^+$: 127.07 (M$^+$, 62%), 112.06 (100%).

2,3,5,6-Tetradeuterio-4-methoxyaniline was diazotised to give the corresponding azide **3** (5.23 mmol, 70%). IR (ν_{max}, neat) 2099 cm^{-1} (N_3); ^{1}H NMR (400 MHz) δ_H 3.79 (s, 3H), ^{13}C NMR (100 MHz) δ_C 55.5 (CH_3), 114.7 (t, J = 24.2 Hz, CD), 119.5 (t, J = 24.9 Hz, CD), 123.1 (C), 156.8 (C); TOF MS CI$^+$: 154.1 (M$^+$ + 1, 25%), 139.09 (5%), 126.09 (100%).

9.5 GHz EPR spectrum of **17a**$^{+\bullet}$ obtained on treatment 3-methoxyphenyl azide **4** with $GaCl_3$ in CH_3CN at 300 K.

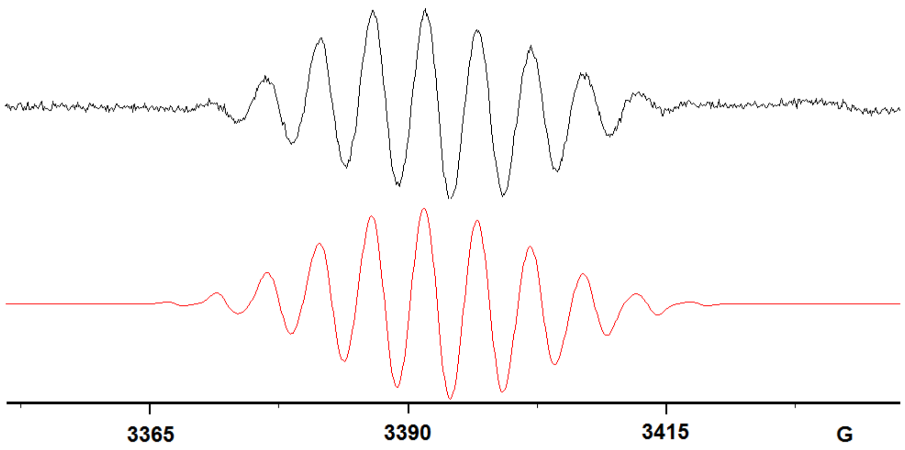

Top: 1st derivative spectrum of $[3\text{-}MeOC_6H_4NHC_6H_4NH_2]^{+\bullet}$ (**17a**$^{+\bullet}$) derived from azide **4**.

Bottom: computer simulation.

9.5 GHz EPR spectrum obtained on treatment 2-methoxyphenyl azide **5** with $GaCl_3/Et_3SiH$ in CH_3CN at 300 K.

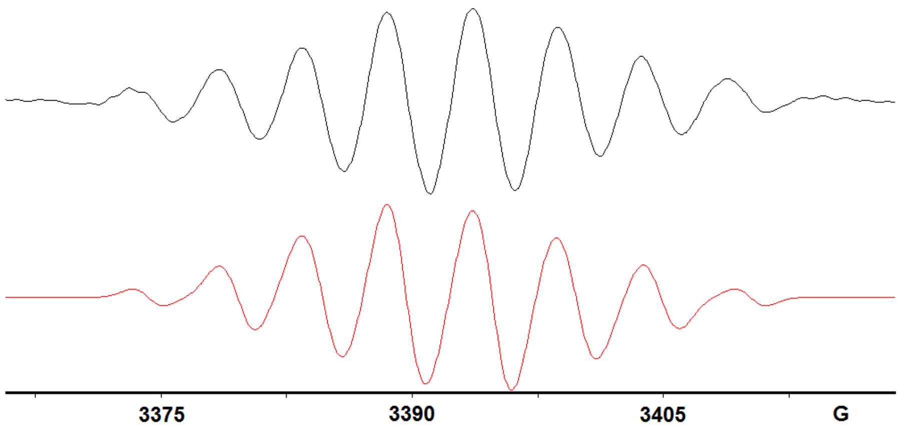

Top: 1st derivative spectrum of $[2\text{-}MeOC_6H_4NHC_6H_4NH_2]^{+\bullet}$ (**17b**$^{+\bullet}$) derived from azide **5**.

Bottom: computer simulation.

Experimental and simulated Davies ENDOR spectrum after the Ga promoted reaction of azide **6** recorded at 50K. Deconvolution of the simulation.

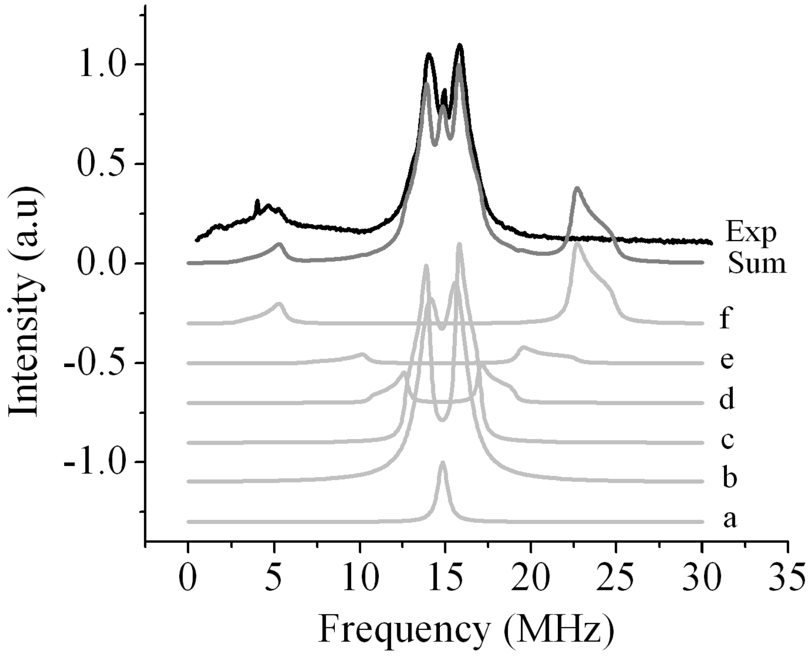

The key to individual traces “a” to “f” is in the table on the following page.

One set of hyperfine coupling parameters derived from the ENDOR spectral simulations[a]

**11c**$^{+\bullet}$ from 4-$FC_6H_4N_3$ (**6**)	**(N)H**	**(N)H_2**	**2H**	**2H**	**2H**	**2H**	**H**	**^{19}F**
A_{iso}	4.0	4.0	2.0	2.0	1.0	0.5	0	6.6
A_{xx}	3.2	3.2	1.5	1.5	0.6	0.4	−0.1	6.1
A_{yy}	3.2	3.2	1.5	1.5	0.6	0.4	−0.1	6.1
A_{zz}	5.5	5.5	3.0	3.0	1.5	0.8	0.1	7.7
Key	e	e	d	d	c	b	a	f

[a]Hfs in Gauss. Species **11c**$^{+\bullet}$ is: $[4\text{-}FC_6H_4NHC_6H_4NH_2]^{+\bullet}$.

DFT computed structures

Computed with the Gaussian 03 suite of programmes using the UB3LYP functional and a 6-31+G(d,p) basis set [1].

$PhNHC_6H_4NH_2$ radical cation **11a**$^{+\bullet}$

Centre number	Atomic number	Atomic type	Coordinates (Å) X	Y	Z
1	6	0	3.170349	-1.442291	0.564745
2	6	0	1.908804	-0.854340	0.620257
3	6	0	1.722761	0.428895	0.075049
4	6	0	2.808636	1.125358	-0.486442
5	6	0	4.062886	0.525317	-0.533322
6	6	0	4.246852	-0.762377	-0.015759
7	1	0	3.318459	-2.426638	0.996896
8	1	0	1.094483	-1.361812	1.124816
9	1	0	2.660583	2.119480	-0.899474
10	1	0	4.896642	1.060221	-0.975941
11	7	0	0.478004	1.090324	0.127409
12	1	0	0.539193	2.098603	0.226754
13	6	0	-0.788958	0.587578	0.052056
14	6	0	-1.876629	1.443073	0.398485
15	6	0	-1.076903	-0.731555	-0.401960
16	6	0	-3.172678	0.998218	0.337645
17	1	0	-1.672378	2.455989	0.734214
18	6	0	-2.375508	-1.175198	-0.469360
19	1	0	-0.273870	-1.376532	-0.735505
20	6	0	-3.459119	-0.331113	-0.089964
21	1	0	-3.986967	1.658833	0.618891
22	1	0	-2.582991	-2.175126	-0.838291
23	7	0	-4.732579	-0.775899	-0.149761
24	1	0	-5.511025	-0.182915	0.100662
25	1	0	-4.949999	-1.713874	-0.455035
26	1	0	5.226938	-1.226603	-0.050096

E = −573.8212633 H

4-$FC_6H_4NHC_6H_4NH_2$ radical cation **11c**$^{+\bullet}$

1	6	0	2.821069	−1.188737	0.590816
2	6	0	1.535241	−0.660750	0.634965
3	6	0	1.278622	0.606463	0.078127
4	6	0	2.329420	1.354377	−0.487054
5	6	0	3.615824	0.831840	−0.530234
6	6	0	3.837649	−0.437714	0.002615
7	1	0	3.049319	−2.155617	1.025126
8	1	0	0.749946	−1.209803	1.141501
9	1	0	2.132645	2.335654	−0.909408
10	1	0	4.438335	1.385005	−0.969345
11	7	0	0.000657	1.198005	0.124455
12	1	0	0.003083	2.207903	0.224657
13	6	0	−1.236345	0.622374	0.049101
14	6	0	−2.370756	1.413241	0.397180
15	6	0	−1.447589	−0.710529	−0.405325
16	6	0	−3.639246	0.894575	0.336242
17	1	0	−2.224998	2.435969	0.733812
18	6	0	−2.718596	−1.228637	−0.472412
19	1	0	−0.609724	−1.308284	−0.741451
20	6	0	−3.848945	−0.448724	−0.092281
21	1	0	−4.490007	1.507210	0.618078
22	1	0	−2.868101	−2.238407	−0.842459
23	7	0	−5.094591	−0.966067	−0.152649
24	1	0	−5.906061	−0.419146	0.097866
25	1	0	−5.257895	−1.914425	−0.459727
26	9	0	5.078102	−0.948480	−0.036859

$E = -673.0574539$

2-$MeOC_6H_4NHC_6H_4NH_2$ radical cation **17b**$^{+\bullet}$

Centre number	Atomic number	Atomic type	Coordinates (Å) *X*	*Y*	*Z*
1	6	0	−1.996012	−2.289568	−0.746464
2	6	0	−0.845302	−1.518080	−0.671920
3	6	0	−0.925556	−0.174270	−0.256571
4	6	0	−2.200787	0.400256	0.037500
5	6	0	−3.350952	−0.389664	−0.045395
6	6	0	−3.243533	−1.727865	−0.424214
7	1	0	−1.933135	−3.319234	−1.081063
8	1	0	0.111552	−1.932385	−0.968548
9	1	0	−4.322622	0.030477	0.182533
10	7	0	0.165678	0.682467	−0.201922
11	1	0	−0.092429	1.663139	−0.287601
12	6	0	1.523303	0.434722	−0.153849
13	6	0	2.371244	1.374818	−0.796805
14	6	0	2.119341	−0.637085	0.607635
15	6	0	3.739350	1.235866	−0.778774
16	1	0	1.910368	2.190984	−1.345764
17	6	0	3.533424	−0.763671	0.582204
18	6	0	4.322880	0.142007	−0.089641
19	1	0	4.366796	1.947956	−1.303013
20	1	0	3.985890	−1.585365	1.129450
21	1	0	−4.140781	−2.335018	−0.484880
22	1	0	5.401180	0.021855	−0.082506
23	7	0	1.398867	−1.480359	1.388382
24	1	0	1.878629	−2.102435	2.022853

25	1	0	0.396374	−1.422784	1.478074
26	8	0	−2.166804	1.706960	0.375850
27	6	0	−3.399774	2.408932	0.627493
28	1	0	−3.913115	1.976630	1.491190
29	1	0	−3.108212	3.434702	0.846474
30	1	0	−4.043636	2.382660	−0.256412

E = −688.34327259

3-$MeOC_6H_4NHC_6H_4NH_2$ radical cation **17a**$^{+\bullet}$

Centre number	Atomic number	Atomic type	Coordinates (Å) *X*	*Y*	*Z*
1	6	0	2.193525	1.687765	−0.870235
2	6	0	0.932180	1.111737	−0.775701
3	6	0	0.820577	−0.187702	−0.222885
4	6	0	1.960427	−0.897195	0.176517
5	6	0	3.222952	−0.305549	0.067741
6	6	0	3.338662	1.003302	−0.451951
7	1	0	2.297242	2.677484	−1.302796
8	1	0	0.062207	1.615880	−1.178524
9	7	0	−0.419350	−0.826957	−0.122226
10	1	0	−0.395554	−1.837851	−0.209962
11	6	0	−1.666113	−0.270425	0.057443
12	6	0	−2.802170	−1.008042	−0.308410
13	6	0	−1.799335	1.008887	0.681597
14	6	0	−4.079939	−0.476817	−0.099510
15	1	0	−2.687256	−1.977411	−0.784663
16	6	0	−3.072947	1.535610	0.876905
17	6	0	−4.202738	0.821579	0.495793
18	1	0	−3.186624	2.501208	1.357900
19	1	0	4.308020	1.475592	−0.550482
20	1	0	−5.192495	1.234772	0.665815
21	1	0	−0.923964	1.521965	1.058816
22	7	0	−5.205199	−1.148953	−0.454503
23	1	0	−6.119738	−0.744225	−0.326235
24	1	0	−5.162718	−2.062756	−0.879067
25	1	0	1.885119	−1.892868	0.602062
26	8	0	4.259234	−1.054515	0.490184
27	6	0	5.591617	−0.523817	0.434575
28	1	0	5.681670	0.372377	1.057232
29	1	0	6.230779	−1.310717	0.832081
30	1	0	5.881087	−0.301955	−0.597829

E = −688.3406428

4-$MeOC_6H_4NHC_6H_4NH_2$ radical cation **11b**$^{+\bullet}$

Centre number	Atomic number	Atomic type	Coordinates (Å) *X*	*Y*	*Z*
1	6	0	−2.357830	−1.165821	−0.590015
2	6	0	−1.068758	−0.671737	−0.580213
3	6	0	−0.812582	0.627131	−0.081816
4	6	0	−1.893178	1.416084	0.363604
5	6	0	−3.189184	0.922283	0.356554
6	6	0	−3.434250	−0.384518	−0.111982

7	1	0	-2.573670	-2.151349	-0.987920
8	1	0	-0.268395	-1.266328	-1.003723
9	1	0	-1.708732	2.419602	0.738075
10	1	0	-3.996562	1.547658	0.715957
11	7	0	0.460926	1.195830	-0.065400
12	1	0	0.469828	2.209960	-0.096176
13	6	0	1.705402	0.610389	-0.010852
14	6	0	2.832243	1.402356	-0.361238
15	6	0	1.921988	-0.721556	0.428933
16	6	0	4.104790	0.883412	-0.318204
17	1	0	2.684284	2.428267	-0.687655
18	6	0	3.196770	-1.241229	0.477022
19	1	0	1.091398	-1.323313	0.775621
20	6	0	4.321100	-0.460763	0.092702
21	1	0	4.950873	1.501192	-0.602955
22	1	0	3.348710	-2.254094	0.837606
23	7	0	5.571522	-0.980077	0.133296
24	1	0	6.378828	-0.429123	-0.117977
25	1	0	5.737852	-1.929569	0.431960
26	8	0	-4.641626	-0.966558	-0.163596
27	6	0	-5.804187	-0.248672	0.277872
28	1	0	-6.637932	-0.933482	0.132096
29	1	0	-5.719924	0.010906	1.338102
30	1	0	-5.955721	0.652079	-0.325578

E = -688.356531

F-trimer radical cation 6-31G(d) **19b**$^{+\bullet}$

Centre number	Atomic number	Atomic type	Coordinates (Å) *X*	*Y*	*Z*
1	6	0	0.170802	1.472474	-0.396222
2	6	0	-1.094857	0.926510	-0.430289
3	6	0	-1.267522	-0.479307	-0.399334
4	6	0	-0.111366	-1.298786	-0.380740
5	6	0	1.154778	-0.755149	-0.341569
6	6	0	1.329784	0.653585	-0.328906
7	1	0	0.284410	2.552385	-0.436298
8	1	0	-1.951446	1.582041	-0.525296
9	1	0	-0.223129	-2.379964	-0.393885
10	1	0	2.017462	-1.409574	-0.344031
11	7	0	-2.502090	-1.096320	-0.450175
12	1	0	-2.477836	-2.065486	-0.747701
13	6	0	-3.773858	-0.610016	-0.166777
14	6	0	-4.883618	-1.273372	-0.741180
15	6	0	-4.012521	0.468604	0.714708
16	6	0	-6.173073	-0.858761	-0.480351
17	1	0	-4.719437	-2.111791	-1.412936
18	6	0	-5.303427	0.885588	0.978163
19	1	0	-3.188817	0.946969	1.230511
20	6	0	-6.416185	0.242072	0.380231
21	1	0	-7.008912	-1.376249	-0.941235
22	1	0	-5.469165	1.702335	1.674365
23	7	0	-7.684991	0.660652	0.635713
24	1	0	-8.482873	0.187506	0.240889
25	1	0	-7.868324	1.427009	1.264493
26	7	0	2.561362	1.251166	-0.307302
27	1	0	2.574483	2.244605	-0.501973
28	6	0	3.828917	0.654951	-0.080050

29	6	0	4.030174	-0.259128	0.966340
30	6	0	4.910385	1.045058	-0.885041
31	6	0	5.294794	-0.801539	1.187147
32	1	0	3.210795	-0.523562	1.626023
33	6	0	6.180708	0.518863	-0.660466
34	1	0	4.755387	1.751294	-1.695473
35	6	0	6.347145	-0.403283	0.368248
36	1	0	5.475078	-1.503983	1.993278
37	1	0	7.028620	0.805265	-1.272470
38	9	0	7.573041	-0.923052	0.585623

E (HF) =-959.4962286

2-$MeOC_6H_4NHC_6H_4NHC_6H_4NH_2$ trimer radical cation **18**$^{+\bullet}$ (6-31G(d))

Centre number	Atomic number	Atomic type	Coordinates (Å) *X*	*Y*	*Z*
1	6	0	1.705037	-2.016794	1.880818
2	6	0	1.265908	-0.801776	1.363281
3	6	0	2.004139	-0.153068	0.365254
4	6	0	3.220448	-0.732346	-0.090410
5	6	0	3.654144	-1.948296	0.439602
6	6	0	2.892064	-2.590853	1.416096
7	1	0	1.129280	-2.507571	2.658717
8	1	0	0.363152	-0.342361	1.748464
9	1	0	4.578918	-2.395768	0.095281
10	7	0	1.677027	1.091897	-0.195136
11	1	0	2.495199	1.556724	-0.579626
12	6	0	0.559266	1.901471	-0.073216
13	6	0	0.766464	3.293691	0.056073
14	6	0	-0.787310	1.415825	-0.183942
15	6	0	-0.295154	4.168793	0.158911
16	1	0	1.787553	3.659816	0.118132
17	6	0	-1.860385	2.332474	-0.067376
18	6	0	-1.618430	3.679584	0.106971
19	1	0	-0.111235	5.230284	0.288148
20	1	0	-2.875566	1.971290	-0.175282
21	1	0	3.233116	-3.539074	1.819114
22	1	0	-2.454084	4.369413	0.167120
23	7	0	-0.991366	0.093129	-0.514705
24	1	0	-0.191966	-0.363101	-0.942256
25	8	0	3.876751	-0.003456	-1.030558
26	6	0	5.162384	-0.447434	-1.478821
27	1	0	5.083152	-1.415437	-1.984956
28	1	0	5.503807	0.308140	-2.186225
29	1	0	5.865258	-0.518014	-0.641750
30	6	0	-2.168882	-0.665723	-0.543071
31	6	0	-2.335252	-1.555567	-1.625901
32	6	0	-3.123850	-0.667009	0.521358
33	6	0	-3.428730	-2.394200	-1.706296
34	1	0	-1.591710	-1.547314	-2.418732
35	6	0	-4.242998	-1.522701	0.402051
36	6	0	-4.395055	-2.365359	-0.681650
37	1	0	-3.547939	-3.056290	-2.557050
38	1	0	-4.984108	-1.514800	1.196779
39	1	0	-5.263735	-3.014004	-0.736140
40	7	0	-2.996592	0.111017	1.642599
41	1	0	-3.556051	-0.123584	2.450861
42	1	0	-2.143368	0.612720	1.836168

E = -974.7058337

References

1. *Gaussian 03,* Revision A.1; Gaussian, Inc.: Pittsburgh, PA, 2003.
